# Hippocampus-dependent learning influences hippocampal neurogenesis

**DOI:** 10.3389/fnins.2013.00057

**Published:** 2013-04-16

**Authors:** Jonathan R. Epp, Carmen Chow, Liisa A. M. Galea

**Affiliations:** Department of Psychology, Program in Neuroscience, Brain Research Centre, University of British ColumbiaVancouver, BC, Canada

**Keywords:** neurogenesis, cell survival, spatial learning, hippocampus, dentate gyrus, memory

## Abstract

The structure of the mammalian hippocampus continues to be modified throughout life by continuous addition of neurons in the dentate gyrus. Although the existence of adult neurogenesis is now widely accepted the function that adult generated granule cells play is a topic of intense debate. Many studies have argued that adult generated neurons, due to unique physiological characteristics, play a unique role in hippocampus-dependent learning and memory. However, it is not currently clear whether this is the case or what specific capability adult generated neurons may confer that developmentally generated neurons do not. These questions have been addressed in numerous ways, from examining the effects of increasing or decreasing neurogenesis to computational modeling. One particular area of research has examined the effects of hippocampus dependent learning on proliferation, survival, integration and activation of immature neurons in response to memory retrieval. Within this subfield there remains a range of data showing that hippocampus dependent learning may increase, decrease or alternatively may not alter these components of neurogenesis in the hippocampus. Determining how and when hippocampus-dependent learning alters adult neurogenesis will help to further clarify the role of adult generated neurons. There are many variables (such as age of immature neurons, species, strain, sex, stress, task difficulty, and type of learning) as well as numerous methodological differences (such as marker type, quantification techniques, apparatus size etc.) that could all be crucial for a clear understanding of the interaction between learning and neurogenesis. Here, we review these findings and discuss the different conditions under which hippocampus-dependent learning impacts adult neurogenesis in the dentate gyrus.

## Introduction

It was previously believed that no new neurons were added to the adult mammalian brain. However, thanks to observations, in both adolescent and in middle aged rats, made by Joseph Altman in the 1960s (Altman and Das, 1965) it was recognized that certain areas of the adult brain, the subventricular zone and the subgranular zone of the hippocampus, continue to produce new neurons throughout life. Now, adult neurogenesis in these areas has been observed in all mammalian species examined including non-human primates and humans (however, see Amrein et al., 2007 for a possible exception in some bat species). Although adult neurogenesis has been seen in other areas of the brain (Gould et al., 1999b; Gould, 2007; Cameron and Dayer, 2008) it is still controversial and it occurs at a relatively low rate compared to neurogenesis in the hippocampus. This review will concentrate on adult neurogenesis in the hippocampus which is now widely accepted.

**Figure 1:**
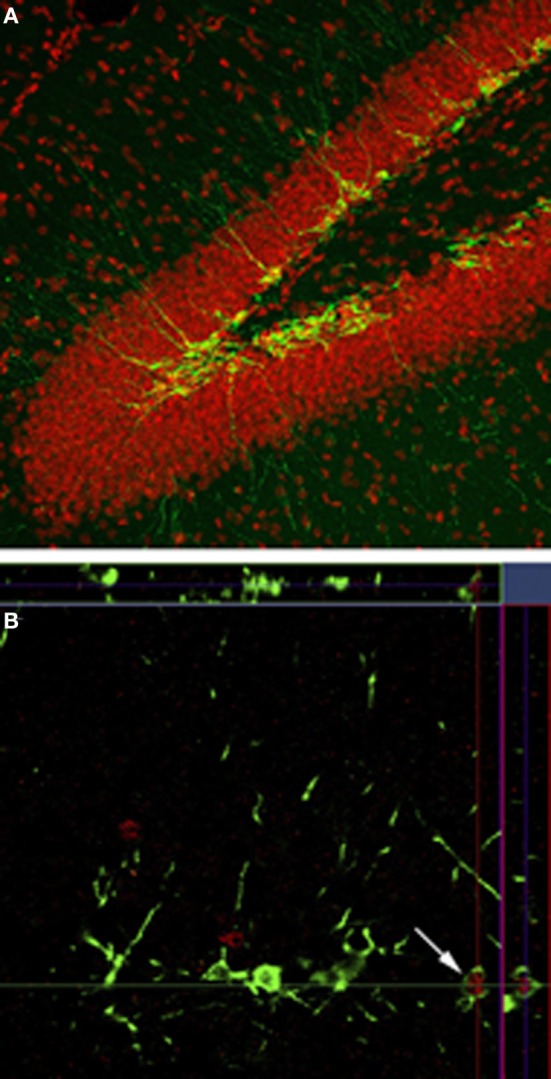
**Neurogenesis in the dentate gyrus. (A)** Doublecortin labeling (green) shows the presence of immature neurons along the inner edge of the granule cell layer and in the subgranular zone. **(B)** Doublecortin (green) and zif268 (red) shows the immature neurons that have been activated in response to spatial memory retrieval.

We will use the term “mature neuron” to refer to granule cells from both developmental and adult origin that no longer possess the characteristics of immature neurons and “immature neuron” to refer to adult generated neurons that have not yet completed their developmental process. Immature neurons can be identified with a variety of labeling strategies (Figure 1). For example endogenous proteins such as doublecortin can be labeled using immunohistochemical techniques. Doublecortin is a protein expressed in immature neurons from the time of cell division until approximately 21 days of age (Brown et al., 2003; Couillard-Despres et al., 2005). Doublecortin expression gives a broad measure of the age of immature neurons but a more precise age can be determined by administering the DNA synthesis marker Bromodeoxyuridine (BrdU). BrdU is incorporated into cells that are in S-phase but is only biologically active for approximately 2 h (Packard et al., 1973; Nowakowski et al., 1989) so it is incorporated into dividing cells only during that time window. Once labeled, BrdU remains incorporated in cells and the number of surviving immature neurons of a particular age can be measured at different times after BrdU administration.

The function of adult neurogenesis in the hippocampus remains a matter of debate. It is possible that adult neurogenesis is merely a developmental byproduct and serves no special function in the adult brain. According to this view, the adult generated neurons serve the same functions as developmentally generated neurons. Others believe that adult neurogenesis is an important mechanism of plasticity in the adult brains and may be related to learning and memory or even emotional or stress regulation (Jacobs et al., 2000; Snyder et al., 2011). Various specific mnemonic functions have been proposed to fall within the special domain of adult generated neurons including, a mechanism for encoding time (Aimone et al., 2006), or pattern separation (Clelland et al., 2009). Still another theory has proposed that adult generated neurons resupply the active pool of neurons in the dentate gyrus while the more mature neurons no longer function (Lisman, 2011). Regardless of what the function may be it is important to note that adult generated neurons are in fact functional (van Praag et al., 2002). Once mature, adult generated neurons exhibit electrophysiological and morphological properties that are practically indistinguishable from developmentally generated neurons. In mice, this maturation process is complete by 4 months of age but possibly as early as 7 weeks (van Praag et al., 2002; Laplagne et al., 2006) although it is important to note that the timing of maturation of immature neurons in the hippocampus is faster in rats than in mice (Snyder et al., 2009a) and is likely different in other species as well.

Adult generated immature neurons do differ from mature neurons in terms of morphological and electrophysiological properties. Beginning as early as 4–10 days after cell division in rats and 10–11 days in mice, immature neurons extend axons into CA3 and dendrites into the molecular layer (Hastings and Gould, 1999; Markakis and Gage, 1999; Zhao et al., 2006). In mice, the growth of these projections and the subsequent formation of synapses continue over a period of several weeks culminating with adult generated neurons that have the same soma size as mature granule cell by 4 months (van Praag et al., 2002; Esposito et al., 2005; Zhao et al., 2006). Initially for a period of 3–4 weeks in mice and rats these immature neurons are highly excitable (Piatti et al., 2006).

The difference in excitability between immature and mature cells in the adult brain recapitulates a phenomenon that occurs during early brain development. During development, the inhibitory transmitter GABA does not exert inhibitory control (Wang et al., 2000). Immature adult generated neurons are also insensitive to inhibition by GABA. In fact, there is evidence that GABA can depolarize immature neurons due to the presence of high levels of the chloride transporter NKCC1 which causes a high internal chloride concentration (Ben-Ari, 2002; Ge et al., 2006). As the cells mature there is a switch in expression from NKCC1 to the chloride exporter KCC2 which causes a decrease in internal chloride concentration and the effect of GABA on the cell becomes hyperpolarization. Thus, for a period of time immature neurons are highly excitable compared to mature neurons and as a result may confer a degree of excitability to a region that is otherwise relatively silent. Long-term potentiation (LTP) is a putative mechanism of associative learning. A specific type of this plasticity, described by Snyder and colleagues (2001), can be induced in hippocampal slices in the absence of GABAergic inhibition. They determined that the unique excitability of immature neurons was responsible for LTP induced without GABAergic inhibition because, either blocking the NR2B subunit of the NMDA receptor (expressed highly during development) or using irradiation to block neurogenesis, prevented the expression of LTP (Snyder et al., 2001). Thus, while mature neurons may not respond to weak stimulation, immature neurons in the dentate gyrus are not under the same type of inhibition and are more likely to be excited. There is further evidence that immature neurons may be preferentially recruited for the storage of hippocampus-dependent learning. A study using the immediate early gene products c-fos and Arc has demonstrated that immature neurons in male mice between 4 and 8 weeks of age are activated in response to spatial memory retrieval (Kee et al., 2007). Similarly, new neurons were activated when spatial training occurred 5 weeks following BrdU injection in male mice (Stone et al., 2011). However, Stone and colleagues (2011) found, that if mice were trained 1 week after 5 days of BrdU injections and examined at 5 weeks, cells were less likely to be activated during memory retrieval, suggesting that one week old neurons are not preferentially incorporated into the spatial memory trace, and that, similar to cell survival, cell activation is also dependent on the age at which learning occurs. A recent study demonstrated that optogenetic silencing of 4 week old newborn neurons in female mice impaired spatial and contextual memory retrieval suggesting that immature neurons of this age are involved in memory retention (Gu et al., 2012). In rats, immature neurons appear to become involved in spatial memory at an earlier time point, as early as 15–20 days of age (Epp et al., 2011a; Snyder et al., 2012). Together, these findings suggest that adult neurogenesis is an important component of hippocampus-dependent learning with different timelines in rats and mice.

Numerous studies have investigated the role of immature neurons in learning and memory by experimentally manipulating levels of neurogenesis. Neurogenesis can be ablated or increased by various techniques prior to learning or memory retrieval in order to examine the impact of adult generated neurons (van Praag et al., 1999; Malberg et al., 2000; Shors et al., 2002; Snyder et al., 2005; Kitamura et al., 2009; Singer et al., 2009). This methodology has been used extensively and has produced a great deal of evidence for the role of adult neurogenesis. We will not discuss these studies here but they have been reviewed elsewhere (Wojtowicz, 2006; Wojtowicz et al., 2008; Deng et al., 2010). Instead, here we will review the existing data on the regulation of neurogenesis in the dentate gyrus by hippocampus-dependent learning and the factors that are known to regulate this relationship.

## Spatial learning modifies survival of immature neurons in the hippocampus

The first demonstration that neurogenesis responded to learning came from Elizabeth Gould and colleagues (Gould et al., 1999a). In this study, BrdU was given to label dividing cells one week prior to training. Male rats trained in the Morris water maze, a spatial learning task that depends on the hippocampus, had a greater number of BrdU-labeled cells in the dentate gyrus. In the young adult rat the rate of cell proliferation is very high relative to the number of immature neurons that survive to maturity. Many of the immature cells die during the first 1–2 weeks (Cameron et al., 1993). However, as Gould and colleagues (1999a) demonstrated, hippocampus-dependent learning was able to rescue these cells and promote their long-term survival and incorporation into the dentate gyrus. This initial study provided compelling evidence of an interaction between learning and adult neurogenesis and supported the possibility of a functional role for adult generated neurons. This result has been supported by a number of studies that also investigated the effects of spatial learning on the survival of immature cells (Ambrogini et al., 2000; Hairston et al., 2005; Epp et al., 2007, 2010, 2011b). However, some studies have produced contradictory findings, either showing that spatial learning decreased the survival of immature neurons (Dobrossy et al., 2003; Ambrogini et al., 2004; Mohapel et al., 2006; Epp et al., 2011a), or that spatial learning had no effect on cell survival (Ehninger and Kempermann, 2006; Mohapel et al., 2006; Van der Borght et al., 2006). The lack of consistent outcomes from the studies described above strongly suggested that although spatial learning can positively influence cell survival, the effect is not a universal one. There must be certain conditions under which cell survival is enhanced and certain conditions under which cell survival is decreased or is not affected. An examination of these studies turns up a variety of methodological factors that could potentially explain the different outcomes, including, age of immature neurons at time of exposure to spatial learning, species/strain differences, sex differences, and strength/difficulty of the training protocol (Epp et al., 2007, 2010, 2011b; Chow et al., in press). Indeed we now know that most of these factors influence the effect of spatial learning on cell survival and these are reviewed here.

## Factors that regulate the effects of spatial learning on cell survival: age of immature neurons on exposure to spatial training

One of the key differences between many of the studies that showed different effects of learning on cell survival was the time course of the experiment or the age of the immature neurons being examined at the time of learning. Gould and colleagues trained their rats on days 7–10 following BrdU injection (day 0) and this scenario led to an increase in cell survival (Gould et al., 1999a). On the other hand, Ambrogini et al. (2004) trained their rats on days 10–14 after BrdU injection and found survival of this population of cells to be decreased. Dupret and colleagues (2007) showed that spatial learning increased cell survival when training occurred 7–12 days after BrdU injection, but at the same time, decreased the survival of cells that were 3 days old at the start of training. Specifically, Dupret and colleagues show that it is the late phase of learning that induces death of 7–9 day old neurons, presumably those that have not received stimulation during training. Taken together, these studies show that the timing of spatial training relative to cell birth is important in determining cell survival. Furthermore, the effect of learning on adult neurogenesis is to selectively stabilize a group of neurons while removing and replacing unused new neurons. A possible interpretation of this is that a critical period exists during the development of immature neurons. This was demonstrated to be true in a study that systematically explored the effects of spatial learning on cell survival during three periods of immature neuron development in the rat (Epp et al., 2007). In this study, rats were trained in the Morris water maze on days 1–5, 6–10, or 11–15 following BrdU administration (day 0) and perfused on day 16. The results showed that cell survival was enhanced only when training occurred during days 6–10 following BrdU injection indicating that this intermediate time period appears to be a critical window during which spatial learning can modulate the survival of immature neurons. This time also corresponds, in rats, to the period when new axons have just reached and are beginning to form connections to area CA3 (Stanfield and Trice, 1988; Hastings and Gould, 1999; Markakis and Gage, 1999). Thus, it is plausible that in order for activity dependent enhancement of cell survival to occur the learning must occur around the time that immature neurons are connecting into the existing circuitry.

**Figure 2:**
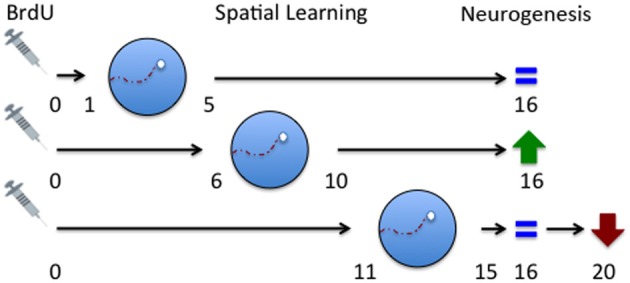
**Critical periods for spatial learning induced changes in immature cell survival in the dentate gyrus.** Spatial learning does not impact the survival of immature neurons that are 1–5 days old at the time of learning. Survival of immature neurons that are 6–10 days old during training is selectively enhanced [although this can depend on task difficulty (Epp and Galea, 2009) and quality of learning (Epp et al., 2007; Sisti et al., 2007)]. Survival of immature neurons that are 15–20 days old at the time of learning is decreased. This effect cannot be detected if animals are perfused the day following training but can be observed if histological examination is delayed until day 20 following a probe trial 90 min before perfusion. Collected from findings from Epp et al. (2007 and 2011a). Described changes in neurogenesis are in comparison to rats trained on a cued version of the task.

**Figure 3:**
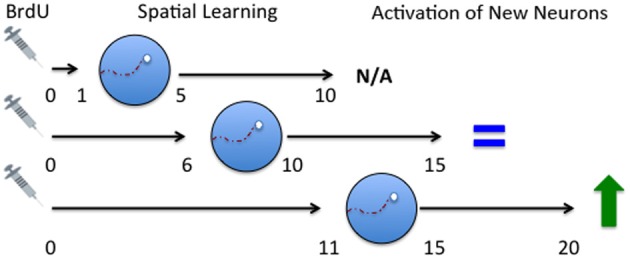
**Time course of activation of immature neurons in response to spatial memory.** Spatial learning occurred either 1–5, 6–10, or 11–15 days following BrdU administration. The rats were then tested with a probe trial 5 days later and were then perfused 2 h later. No activation was seen in 10-day-old neurons. Rats trained on the spatial version of the task on days 6–10 had a small percentage of 15 day old neurons were activated but no difference existed between rats that received spatial versus non-spatial training (Epp et al., 2011a). Rats trained the spatial version of the task on days 6–10 (Chow et al., in press) or 11–15 showed enhanced activation of 20 day old neurons compared to rats that were trained on the non-spatial version of the task (Epp et al., 2011a). N/A, no activation; IEG, immediate early gene. Described changes in activation are in comparison to rats trained on a cued version of the task.

Based on the idea of critical periods to influence survival of immature neurons after exposure to spatial learning and the results of the (Ambrogini et al., 2004) study, we predicted that training on days 11–15 should have resulted in a decrease in cell survival but no significant change in cell survival was observed in our study (Epp et al., 2007). A key difference between the Epp et al. (2007) and the Ambrogini et al. (2004) study was the timing of perfusion AFTER spatial training. Epp et al. (2007) perfused rats 24 h after training on days 11–15 after BrdU injection while Ambrogini et al. (2004) waited 3 days after training to perfuse the rats. In a follow up study we trained rats on days 11–15 and perfused them 5 days following training, or on day 20 following BrdU administration (Epp et al., 2011a). Confirming their results, we showed that immature neuron survival was decreased by spatial learning using this paradigm which more closely conformed to the original Ambrogini study (2004). These results suggest that late training occurring after the critical (6–10) window may decrease neuron survival possibly due to competitive integration of the 6–10 day old neurons. Although the population of cells being examined was approximately 11 days old at the start of training there was also an un-labeled population of cells that were 6–10 days old, the survival of which was likely increased by spatial learning. The 11–15 day old population may lose the competition because they fall outside the critical age and are therefore gradually removed. The loss of these older cells may not be evident immediately after training but may be detected a few days later. This hypothesis fits nicely with a study which demonstrated that survival of immature neurons is dependent on activation of the immature cells and that there is a competitive process that occurs among cells (Tashiro et al., 2006). Furthermore, they showed that the death of cells that do not receive NMDA-receptor activation occurred at about 18 days, similar to the spatial learning studies (Ambrogini et al., 2004; Epp et al., 2011a). These studies further suggest that there is a critical time window for spatial learning to increase cell survival 6–10 days after birth but also show that there is another time window 11–15 days after birth for spatial learning to decrease cell survival (Figure 2). The population of cells that are rescued by spatial learning are also activated later on by spatial memory retrieval suggesting that these immature neurons are part of the memory trace (Figure 3). If rats were trained in the Morris water maze 11–15 days after BrdU injection and given a probe trial on day 20, there was a significant increase in the percentage of BrdU-labeled immature neurons that were co-labeled with c-fos (Epp et al., 2010). Furthermore, the co-expression of BrdU and c-fos correlated positively with the strength of the spatial memory. Several other studies have also shown that spatial learning increases the activation of immature neurons (Snyder et al., 2009b, 2011; Epp et al., 2011a; Chow et al., in press). There are regional differences in activation of immature neurons within the hippocampus. Immature neurons in the ventral dentate gyrus, specifically in the suprapyramidal blade, are activated more readily by spatial learning (Snyder et al., 2009b), although we have also shown that immature neurons in the dorsal dentate gyrus are more activated in response to spatial memory retrieval when using a different training paradigm (Chow et al., in press). Recently, it has also been demonstrated in rats that immature neurons in the septal pole of the dentate gyrus become activated by stimulation at a younger age than immature neurons in the temporal pole (Snyder et al., 2012). These studies demonstrate the importance of segmentation of data across different regions of the dentate gyrus in order to observe more specific changes in cell survival and activation.

## Factors that regulate the effects of spatial learning on cell survival: task differences/difficulty

In addition to spatial learning, training on other hippocampus dependent tasks also enhances the survival of immature neurons. A number of studies have shown that trace eyeblink conditioning can enhance cell survival, at least under certain conditions (Gould et al., 1999a; Shors et al., 2002; Leuner et al., 2006). Tracey Shors and colleagues have shown that the rate of acquisition of the trace eyeblink task is critical for enhancing survival. Faster acquisition was related with increased cell survival while slower acquisition did not result in a significant increase in cell survival (Waddell and Shors, 2008). Importantly, the increase in cell survival following trace eyeblink conditioning appears to occur during the same critical period as during spatial learning. Anderson and colleagues treated rats with BrdU, either 30 min, 1 week or 3 weeks before trace conditioning. An increase in cell survival was found only at the 1 week time point (Anderson et al., 2011). In contrast with our spatial learning studies (Epp et al., 2007, 2010) cell survival was decreased when BrdU was administered just prior to learning. Another hippocampus-dependent task, social transmission of food preference, also increases the survival of immature cells that are 1 week old at the time of learning (Olariu et al., 2005). However, survival was only enhanced after a single, but not multiple, training trials.

In addition to the type of task used, variables that alter the difficulty of a given task can also change how learning influences neurogenesis. In rats trained in the Morris water maze within the 6–10 day time window with four trials per session and ample distal cues in the environment cell survival is increased. However, when the number of trials was reduced to 2 per day cell survival was no longer enhanced (Epp et al., 2010). This procedure slowed learning due to an increase in task difficulty and/or changing the demands of the task such that it may have become dependent on other brain regions. Furthermore, when training took place in an environment with few distal cues, learning became more difficult to achieve and the survival of 6–10 day old cells is decreased. In addition, a more difficult spatial working memory task appears to decreases neurogenesis in comparison to the more standard reference memory version on the Morris water maze (Xu et al., 2011). Trace eyeblink conditioning also has different effects on cell survival as a result of different task demands. Tracey Shors and colleagues demonstrated that spaced trials produces stronger memory and a greater increase in cell survival compared to massed training for trace conditioning (Sisti et al., 2007). This could be a result of task difficulty, type of training or a result of the quality of learning. In a subsequent study they also demonstrated that interfering with learning in order to slow the rate of acquisition is associated with a greater enhancement in cell survival but only in good and not poor learners in trace conditioning (Dalla et al., 2007; Curlik and Shors, 2011). Interestingly, we have observed that cell survival was increased in the Morris water maze in poor learners but not in good learners (Epp et al., 2007). Although there appears to be an interesting interaction between quality of learning and cell survival it is not yet clear how these factors interact, and further study is warranted as it appears that the type of task (trace conditioning or spatial learning) may interact with these factors.

## Factors that regulate the effects of spatial learning on cell survival: sex differences

There are sex differences in cognition as well as adult hippocampal neurogenesis. For example, the most widely reported sex difference in both the human and animal literature is that males outperform females in spatial tasks (Williams et al., 1990; Galea and Kimura, 1993; Galea et al., 1996; Gron et al., 2000; Beiko et al., 2004; van Gerven et al., 2012). Optimal performance in spatial tasks, such as the Morris water maze, requires the integrity of the hippocampus (Morris et al., 1990). Interestingly, the hippocampus is activated in different extents in men and women during spatial tasks, and this sex difference is dependent on the menstrual cycle. Indeed, imaging studies show that in men, the hippocampus is more active during mental rotation (Butler et al., 2006) and spatial navigation tasks (Gron et al., 2000) compared to women. Furthermore, the menses phase alters both spatial ability and activation levels as measured using fMRI in women. During the menses phase (a period of reduced ovarian hormone levels), women performed better on the spatial rotation test, and their activation levels when performing spatial rotation tasks were more closely patterned to the male response compared to women in the midluteal phase (Hampson, 1990; Dietrich et al., 2001).

Sex differences in neurogenesis levels in the hippocampus have also been reported (Galea and McEwen, 1999; Tanapat et al., 1999). Galea and McEwen found that there were sex differences in cell proliferation favoring males during the breeding season (when gonadal hormone levels are elevated), but not during the non-breeding season, in wild meadow voles, suggesting that gonadal hormones mediate the sex difference in cell proliferation. Tanapat and colleagues (1999) found that proestrous females, with elevated estradiol levels, showed greater levels of cell proliferation compared to males and non-proestrous females. Additionally, when females were injected with BrdU during proestrus, they showed significantly higher levels of cell survival compared to males and non-proestrous females for up to 14 days after injection. Interestingly, studies are more equivocal in mice, as one study did not find sex or estrous cycle differences on neurogenesis in the dentate gyrus in mice (Lagace et al., 2007) but other studies do (Ma et al., 2012; Roughton et al., 2012), perhaps due to strain differences. Therefore, gonadal hormone level, timing of BrdU injection and tissue examination, and the animal species or strain are important methodological considerations when examining sex differences in adult hippocampal neurogenesis.

To our knowledge only two studies have directly examined how sex affects the relationship between hippocampus-dependent learning and neurogenesis, and the first study was conducted by Dalla and colleagues (2009). Using the trace eyeblink conditioning task, the authors showed that female rats learned the task faster and also showed a greater increase in cell survival compared to male rats. A second study used a task that favored learning in males, the Morris water maze, and produced the opposite pattern of results showing that male rats outperformed female rats during spatial training and subsequently showed increased cell survival compared to female rats (Chow et al., in press).

In both of these studies (Dalla et al., 2009; Chow et al., in press), the sex differences in learning performance were only observed in the early phases of training. Therefore, in both of these studies, sex differences in performance during the initial acquisition stage corresponded to the direction of the sex difference in cell survival. Due to the similar levels of task mastery in both studies, as reflected by a lack of sex difference in performance toward the end of training, the relationship between learning and neurogenesis may be mediated by sex differences in learning strategy rather than learning ability. For instance, during spatial navigation tasks, males generally attend to geometric/spatial cues (e.g. relative distance between extramaze cues and the hidden platform), which is a strategy that engages the hippocampus. In contrast, females tend to focus on landmark cues, which is a more striatum-dependent strategy (Williams et al., 1990; Galea and Kimura, 1993; McDonald and White, 1994; Miranda et al., 2006). Therefore, the extent to which the hippocampus is activated during learning, as mediated via strategy choice, could influence neurogenesis. It is also possible that sex differences in sensitivity to certain aspects of a task could indirectly influence learning and neurogenesis. For instance, females, but not males, show elevated levels of the stress hormone, corticosterone, after one Morris water maze trial, an effect associated with poorer spatial performance relative to males (Beiko et al., 2004). This sex difference in performance, however, disappeared when animals were given the chance to acclimatize to the task apparatus prior to training. Therefore, it may be that alterations to task procedures that abolish the sex difference in learning performance may alter or even abolish the sex difference in neurogenesis, and would be an interesting point of investigation in future studies.

Intriguingly, activation of immature 20-day old neurons (quantified by co-labeling BrdU with the IEG product zif268) in the dorsal dentate gyrus during spatial memory retrieval was positively correlated with spatial performance during training in females, but not males (Chow et al., in press). Additionally, McClure and colleagues showed that estradiol significantly increased activation of immature neurons in females relative to the control group (McClure et al., 2012). Thus it would be interesting to further investigate the sex differences in activation patterns of younger versus older neurons during spatial learning and how those differences relate to adult neurogenesis.

## Factors that regulate the effects of spatial learning on cell survival: strategy differences

Studies in humans found that females chose to use spatial strategies at least as often as males, but were less adept in strategy execution (Galea and Kimura, 1993; van Gerven et al., 2012). Previous studies in our laboratory using a cue competition paradigm have shown that the same learning strategy can have sexually dimorphic effects on neurogenesis. In the cue competition task, rats are trained to both locate a hidden platform using spatial strategies and locate a visible platform using cue strategies. During the probe trial, the platform is visible and moved to a new quadrant opposite the old platform location, and strategy preference is elucidated based on whether the rat swims to the new location (cue strategy preference) or to the old location (spatial strategy preference). In males, animals that favored the spatial strategy showed a reduction in cell proliferation compared to cue strategy users, while in females, the opposite was true (Epp and Galea, 2009; Rummel et al., 2010). Furthermore, studies in mice have shown that proteins that regulate neurogenesis, such as Cdk5 (Jessberger et al., 2008; Lagace et al., 2008) and the cAMP responding element binding (CREB) protein (Dworkin and Mantamadiotis, 2010), can differentially facilitate or impair the acquisition of hippocampus-dependent tasks such as the Morris water maze (Ris et al., 2005; Hebda-Bauer et al., 2007) and contextual fear conditioning (Kudo et al., 2003) in males and females. Therefore, the same type of learning paradigm may influence neurogenesis in the hippocampus through different mechanisms in males and females.

## Factors that regulate the effects of spatial learning on cell survival: strain/species differences

The majority of the studies examining cell survival following spatial learning were conducted in rats (Gould et al., 1999a; Ambrogini et al., 2004, 2000; Epp et al., 2007, 2011a). Given the tremendous increase in the popularity of mice as a model system it is important to consider whether this effect is similar in rats and mice. A notable exception to the common use of rats was a study conducted by Ehninger and Kempermann (2006) that used female C57Bl/6 mice. In this study, although spatial learning occurred during the 6–10 day time period, a critical window in rats, there was no change in cell survival. Therefore, it is possible that either spatial learning does not have the same effect on cell survival in mice that it does in rats, that the time period during which survival may be enhanced is different or that, as we have shown in rats, females do not show the same increase in cell survival with spatial learning (Chow et al., in press). There is some supporting evidence that either of these theories may be true. Recently it has been demonstrated that compared to rats, adult generated neurons in mice mature more slowly and do not appear to be as important to hippocampal function (Snyder et al., 2009a). Further, spatial training caused a greater increase in the proportion of immature neurons that expressed the immediate early gene product zif268 in rats compared to mice. Additionally, abolishing neurogenesis in the dentate gyrus with irradiation impaired fear conditioning in rats but not mice (Snyder et al., 2009a).

It should also be pointed out that exposure to a complex environment can cause a similar increase in survival of immature neurons as shown by Tashiro and colleagues (Tashiro et al., 2007). However, the critical window in that study occurred between 2 and 3 weeks after cell division, slightly later than seen with spatial learning (1–2 weeks). It is possible that the later critical window is a result of the task used or it may have been because mice were used.

Within species there are numerous strains of both rat and mice that are commonly used and not all have similar neurogenic responses to spatial learning. For example, Long-Evans and Sprague-Dawley rats show similar increases in BrdU-labeled cells following spatial learning. However, when examining the maturation rate of immature neurons following spatial learning Sprague-Dawley rats show an increased percentage of doublecortin labeled cells with a mature phenotype compared to Long-Evans rats (Epp et al., 2011b) This suggests that spatial learning had a strain dependent effect of the rate of neuronal maturation in addition to a more generalized effect on cell survival. In addition, despite having equal levels of doublecortin-labeled neurons in untrained rats, Sprague-Dawley rats showed an increase in doublecortin following spatial learning while Long-Evans rats did not. In mice, baseline differences in neurogenesis do exist across various strains (Kempermann and Gage, 2002) and as a result it stands to reason that many strains may show different regulation of neurogenesis by learning. Although little else is known about strain differences in the response of neurogenesis to spatial learning, there is evidence that different strains respond differently to other treatments such as chronic mild stress. A recent study showed that Lewis rats, characterized by a hypoactive hypothalamus-pituitary-adrenal response, showed an increase in doublecortin labeling following chronic mild stress while Sprague-Dawley and Fischer 344 rats did not (Wu and Wang, 2010).

### The function of learning-induced adult neurogenesis

The role of new neurons that are rescued by learning is still largely unexplored. Based on studies using immediate early genes as a marker for cell activation, new neurons that are approximately 4–10 weeks old in mice (Kee et al., 2007; Stone et al., 2011; Gu et al., 2012) and 16–20 days old in rats (Epp et al., 2011a) appear to be involved in memory retrieval, provided that learning occurred at the critical stage in cell development (at least 4 weeks of age in mice; 11–15 days in rats). This age-dependent incorporation of cells into the memory trace may be due to the fact that prior to the critical age, cells have not yet formed the appropriate connections necessary for processes related to memory consolidation, such as LTP. Indeed, Bruel-Jungerman and colleagues (2006) found that, in rats, LTP is not induced in cells that are less than 2 weeks of age, and in mice, new neurons do not begin to receive synaptic input until approximately 2 weeks of age (Esposito et al., 2005). Interestingly, neurons that are 1 week old at the time of learning have been found to remain in the circuitry for up to 60 days after training in rats (Leuner et al., 2004). Further studies to examine the electrophysiological properties of new neurons at various stages of maturity during learning may provide more definite answers. It is important to keep in mind that when comparing studies in mice and rats Snyder and colleagues have demonstrated that new neurons are more likely to involved with behavior in rats than mice. Future studies examining the contributions of adult generated neurons to hippocampal as well as brain wide network dynamics will be crucial to determine the precise functional contributions of adult neurogenesis.

## Conclusions

Hippocampus-dependent learning can modify the survival of adult generated neurons although this relationship is a complicated one as several important factors and critical time windows that must be considered. Perhaps the most critical factor to consider when examining the effects of spatial learning on neurogenesis in the hippocampus is the effect of the age of the immature neurons at the time of learning. In rats, learning that occurs approximately one week after training shows the greatest potential to increase cell survival. Within this time window, it also appears critical for the learning to proceed with a steep learning curve. Furthermore there exists at least one other critical time period, 11–15 days when learning must occur to see a decrease in cell survival. The difficulty of the task, quality of learning, species strain and sex being tested must also be taken into consideration. Stronger relationships between behavior and neurogenesis exist in rats compared to mice and in male, compared to female, rats for spatial learning. However, when learning does increase the survival of immature neurons, they can be activated by spatial memory retrieval suggesting that they are an important part of the spatial memory trace. Future experiments aimed at understanding how and why spatial learning increases cell survival should attempt to discover a unified framework of the conditions that control this relationship.

### Conflict of interest statement

The authors declare that the research was conducted in the absence of any commercial or financial relationships that could be construed as a potential conflict of interest.
